# Comparative Genome Characterization of a Petroleum-Degrading *Bacillus subtilis* Strain DM2

**DOI:** 10.1155/2019/7410823

**Published:** 2019-05-08

**Authors:** Shi-Weng Li, Meng-Yuan Liu, Rui-Qi Yang

**Affiliations:** ^1^School of Environmental and Municipal Engineering, Lanzhou Jiaotong University, Lanzhou 730070, China; ^2^Key Laboratory of Extreme Environmental Microbial Resources and Engineering in Gansu Province, Lanzhou 730070, China

## Abstract

The complete genome sequence of *Bacillus subtilis* strain DM2 isolated from petroleum-contaminated soil on the Tibetan Plateau was determined. The genome of strain DM2 consists of a circular chromosome of 4,238,631 bp for 4458 protein-coding genes and a plasmid of 84,240 bp coding for 103 genes. Thirty-four genomic islands coding for 330 proteins and 5 prophages are found in the genome. The DDH value shows that strain DM2 belongs to *B. subtilis* subsp*. subtilis* subspecies, but significant variations of the genome are also present. Comparative analysis showed that the genome of strain DM2 encodes some strain-specific proteins in comparison with *B. subtilis* subsp*. subtilis* str. 168, such as carboxymuconolactone decarboxylase family protein, gfo/Idh/MocA family oxidoreductases, GlsB/YeaQ/YmgE family stress response membrane protein, HlyC/CorC family transporters, LLM class flavin-dependent oxidoreductase, and LPXTG cell wall anchor domain-containing protein. Most of the common strain-specific proteins in DM2 and MJ01 strains, or proteins unique to DM2 strain, are involved in the pathways related to stress response, signaling, and hydrocarbon degradation. Furthermore, the strain DM2 genome contains 122 genes coding for developed two-component systems and 138 genes coding for ABC transporter systems. The prominent features of the strain DM2 genome reflect the evolutionary fitness of this strain to harsh conditions and hydrocarbon utilization.

## 1. Introduction

Petroleum exploitation and utilization have caused a widespread distribution of hydrocarbons in the environment. Petroleum comprises alkanes, aromatic hydrocarbons, and nonhydrocarbon compounds. These compounds pose a threat to the ecosystem and human health [1]. Many microbes have been described by their ability to degrade petroleum hydrocarbons and have been used in the bioremediation of petroleum-contaminated environment. However, the microbes inhabiting petroleum-contaminated environments at low-temperature and higher-altitude biotope remain to be studied and exploited.

*Bacillus subtilis*, an extensively studied gram-positive model bacterial species in the *Bacillus* genus, has been isolated from a variety of distinct environments, such as diverse soils and waters [2, 3], fermented foods [4], marine sand [5], rumen and intestinal tract [6], and plant endophytic bacteria [7, 8]. The versatile physiological functions of this bacterium have been explored for industrial production [9], bioremediation of polluted environment [10–12], plant growth promotion and pathogen control [7], and even for use as probiotics for humans [13]. The diverse habitats of these strains reflect versatile metabolic pathways and a robust capacity of environmental adaptation of a widely distributed species [3, 14]. Recently, comparative genomics have shown the significant genetic variations among *B. subtilis* strains that inhabit diverse environments [3, 14–17]. Thus, the ecophysiological diversity of this species provides an ideal model for revealing its genetic and molecular basis of successful environmental adaptability [14].

Recently, several *Bacillus* strains had been isolated from petroleum-contaminated soils and were explored to degrade hydrocarbon compounds, such as *B. subtilis* A1 [18], *Bacillus* sp. M3 [19], and *Bacillus* sp. Q2 [20]. In addition, genome sequences of two *B. subtilis* strains are available. *B. subtilis* strain B-1, which was isolated from an oil field, can form a thick biofilm with an extracellular matrix consisting mainly of gamma-polyglutamate [21]. The B-1 genome displays 50% sequence homology with that of the model laboratory strain *B. subtilis* 168. Another *B. subtilis* strain, MJ01, was isolated from oil-contaminated soil and evaluated as a new biosurfactant-producing strain [12]. Digital DNA-DNA hybridization showed the most similarity (94.7%) with the genome of *B. subtilis* subsp*. spizizenii* TU-B-10. In this study, we isolated a new *B. subtilis* strain from petroleum-contaminated soil on the Tibetan Plateau in China. To further understand its genetic traits for hydrocarbon degradation and adaptability to low-temperature environment, we analyzed the whole genome sequence and compared it with the genomes of other *B. subtilis* strains representing distinct ecotypes or physiological traits. Our aim was to reveal the ecological fitness associated with microbial survival strategies that are relevant to petroleum-contaminated and low-temperature soil environments.

## 2. Materials and Methods

### 2.1. Strain Isolation and Measurement of Petroleum Degradation

*B. subtilis* strain DM2 was isolated from oil field soils in the town of Huatugou, which is located in the northwest of Qinghai province of China (90.71°E, 38.29°N, 2907 m). The strain was isolated and cultured in MM medium (3.5 g/L MgCl_2_, 1.0 g/L NH_4_NO_3_, 0.35 g/L KCl, 0.05 g/L CaCl_2_, 1.0 g/L KH_2_PO_4_, 1.0 g/L K_2_HPO_4_, 0.01 g/L FeCl_3_, 0.08 g/L KBr, 1 × 10^−4^ g/L ZnSO_4_·7H_2_O, and 24 mg/L SrCl_2_·6H_2_O, pH 7.5), with 2% (*v*/*v*) petroleum as a sole carbon source. To assess petroleum degradation, cells were inoculated in 100 mL liquid MM medium with 2% (*v*/*v*) petroleum and cultured on a rotary shaker at 20°C and 150 rpm. After 96 h of fermentation, the residual petroleum in the medium was extracted using petroleum ether. The extraction was subsequently evaporated in a rotary evaporator at 40°C and the amount of residual oil was measured using the gravimetric method described by Latha and Kalaivani [22], i.e., amount of petroleum degraded = weight of petroleum added in the medium − weight of residual oil, and the degradation rate was consequently calculated.

### 2.2. DNA Extraction and 16S rRNA Gene Sequencing

For DNA extraction, the strain was inoculated in liquid LB medium at 25°C and incubated at 150 rpm on a rotary shaker for 60 h. Genomic DNA was extracted using a Bacterial Genomic DNA Extraction Kit (AxyPrep, Corning Inc., NY, USA) according to its instructions. The 16S rRNA gene sequence was amplified using the primers 27F 5′-AGAGTTTGATCCTGGCTCAG and 1492R 5′-TACCTTGTTACGACTT [23]; the sequence was aligned with the NCBI database, and the 16S rRNA gene sequence obtained in this study was deposited into NCBI under accession number MK014304.

### 2.3. Genomic DNA Sequencing, Assembly, and Annotation

The PacBio genomic DNA library was prepared using TruSeq Nano DNA LT Library Preparation Kits (Illumina Inc., San Diego, CA, USA) after purification of the strain DNA and examination using a Nanodrop 2500. The DNA library sequencing was performed on a PacBio RS II platform using Illumina MiSeq at Majorbio Inc. (Shanghai, China). After quality control of the raw reads generated from sequencing, the resulting clean reads were assembled de novo using Newbler (version 2.8) and Hierarchical Genome Assembly Process (HGAP) version 3.0. The protein-coding genes, tRNA genes, and rRNA genes within the genomic sequence assembled were predicted using Glimmer 3.02 (http://www.cbcb.umd.edu/software/glimmer/), tRNAscan-SE v1.3.1, and Barrnap 0.4.2, respectively. The tandem repeat and interspersed repeat sequences were predicted using RepeatMasker and TRF software, respectively. The predicted protein-coding genes were subjected to BLASTn against the Nr, string (v9.05), and GO databases using BLAST2.2.28+. The COG (Clusters of Orthologous Groups of proteins) annotation of the predicted genes was obtained by BLASTp search against the string database (http://string-db.org/), and the functional protein clustering was performed according to the COG results. The predicted genes were further compared by blast against KEGG (Kyoto Encyclopedia of Genes and Genomes) database to gain their KOs and pathways. Genomic Island (GI) in the genome was predicted using IslandViewer 4 (https://www.pathogenomics.sfu.ca/islandviewer/) and PHAST software (version 1.5). The complete genome sequences generated in the present study were deposited in GenBank under the accession numbers CP030937 and CP030938.

### 2.4. Phylogenetic Analysis of the Strain

The protein sequences of 24 housekeeping genes, including CTP synthase, DNA primase, DNA-directed RNA polymerase beta-subunit, LSU ribosomal protein L3p, LSU ribosomal protein L4p, LSU ribosomal protein L5p, LSU ribosomal protein L6p, LSU ribosomal protein L7/L12, LSU ribosomal protein L11p, LSU ribosomal protein L13p, LSU ribosomal protein L16p, LSU ribosomal protein L20p, LSU ribosomal protein L27p, phosphoglycerate kinase, ribosome recycling factor, SSU ribosomal protein S2p, SSU ribosomal protein S3p, SSU ribosomal protein S5p, SSU ribosomal protein S9p, SSU ribosomal protein S10p, SSU ribosomal protein S11p, SSU ribosomal protein S13p, transcription termination protein NusA, and translation elongation factor Ts, from certain *Bacillaceae* members were downloaded from GenBank [13]. The protein sequences extracted from GenBank and the present isolate were aligned using MEGA 7.0, and a phylogenetic tree was consequently produced based on neighbor-joining method.

### 2.5. Comparative Genomics

To discern the characteristic of DM2 genome, the genomes of six *Bacillus* strains, i.e., *B. subtilis* subsp*. subtilis* str. 168, *B. subtilis* PY79, *B. subtilis* TO-A JPC, *B. subtilis* MJ01, *B. subtilis* B-1, *B. subtilis* TO-A JPC, and *B. subtilis* UD1022, which were isolated from different biotopes with their genome sequences deposited in GenBank, were retrieved from NCBI. The genome of strain DM2 was submitted to the Integrated Microbial Genomes (IMG) database (https://img.jgi.doe.gov/) for comparative genome analysis.

## 3. Results and Discussion

### 3.1. Isolation and Identification of a Petroleum-Degrading Strain DM2

**Figure 1:**
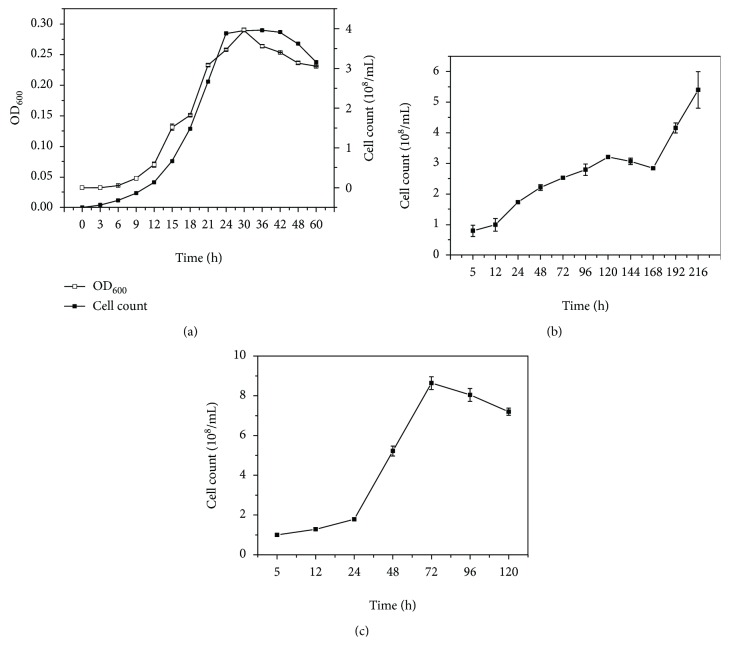
The growth features of strain DM2 in different medium. (a) Growth curve in LB medium. (b) Growth curve in MM medium with the mixture of alkanes (C_12_ : C_14_ : C_15_ = 1 : 1 : 1) as the sole carbon source. (c) Growth curve in MM medium with 2% petroleum as the sole carbon source.

Strain DM2 was isolated from the soil of an oil field located in a cryogenic region at an altitude of 2909 m using MM medium with petroleum as the sole carbon source. The strain grew well in liquid LB medium and reached its maximum growth rate after 24 h of shaking culture (Figure 1(a)). The strain could also grow in the oligotrophic liquid MM medium containing 2% (*v*/*v*) of the mixture of alkanes (C_12_ : C_14_ : C_15_ = 1 : 1 : 1) as the sole carbon source (Figure 1(b)). However, when 2% petroleum was added to MM medium as the sole carbon source, the strain exhibited better growth than with the alkane mixture as the carbon source (Figure 1(c)), suggesting a low degradation capacity for middle-chain alkanes. Further experiments indicated that, when the strain incubated in liquid MM medium containing 2% petroleum as the carbon source at 20°C for 96 h, 53.92%±4.74 of petroleum in medium was degraded suggesting its strong petroleum-degrading ability at the culture conditions. The 16S rRNA gene sequence of strain DM2 showed 99% similarity with *Bacillus subtilis*. Thus, the isolate was identified as *B. subtilis* DM2.

### 3.2. The Genome Organization of *B. subtilis* DM2

**Figure 2:**
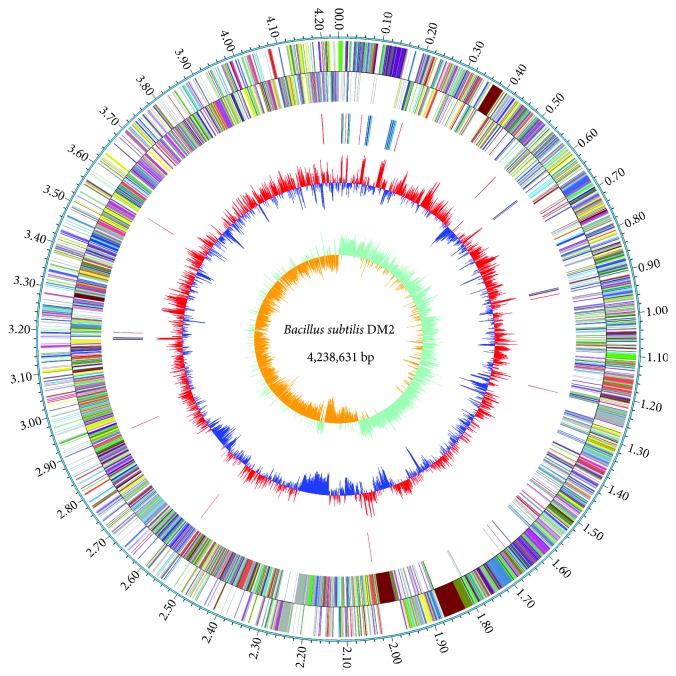
Circular chromosome genome of *B. subtilis* DM2. Circle 1 (from outside to inside) represents the genome size. Circle 2 represents the protein-coding genes in the positive strand of chromosome. Circle 3 represents the protein-coding genes in the negative strand of chromosome. Circle 4 represents rRNA and tRNA genes. Circle 5 represents G+C content, <average content (blue-colored), ≥average content (red-colored). Circle 6 represents the GC skew (G-C)/(G+C).

The genome of strain DM2 consists of a circular chromosome of 4,238,631 bp with G+C content of 43.52% and a plasmid of 84,240 bp with G+C content of 35.08%. The detailed information on the genome is summarized in Table 1 and Figure 2. To further discern the characteristics of the genome, we downloaded the genomic data of six *B. subtilis* strains from the NCBI database and comparatively analyzed their genomes (Table 2). Of those, *B. subtilis* subsp*. subtilis* str. 168 is a subspecies and a model strain of *B. subtilis*. *B. subtilis* PY79 is one of the most widely used laboratory strains [24]. *B. subtilis* B-1 is a petroleum-degrading and biofilm-producing strain isolated from the oil field biofilms [21]. *B. subtilis* MJ01 is also a petroleum-degrading strain isolated from oil-contaminated soil [12]. *B. subtilis* TO-A JPC is a probiotic strain isolated from a probiotic drug Vibact® [13]. *B. subtilis* UD1022 is a plant growth-promoting strain isolated from the plant rhizosphere soil [7]. Among them, DM2 has the largest genome and the highest number of predicted genes and protein-coding genes. The previous studies hypothesized that there is a correlation between microbial genome size and their environment adaptability [25, 26]. Whether such distinct genetic traits of strain DM2 is related to its successful adaptation to its habitat needs further study.

### 3.3. Functional Protein Classification

The genome of strain DM2 encodes 4458 proteins, of which 712 are hypothetical proteins. The predicted protein sequences were aligned against the COG database using BLASTp. A total of 3163 proteins were annotated to at least one COG category. The top protein categories are amino acid transport and metabolism, carbohydrate transport and metabolism, general function prediction only, transcription, function unknown, translation, ribosomal structure and biogenesis, coenzyme transport and metabolism, cell wall/membrane/envelope biogenesis, inorganic ion transport and metabolism, signal transduction mechanisms, and energy production and conversion (Table S1). The protein numbers under categories of extracellular structures, lipid transport and metabolism, secondary metabolite biosynthesis, transport and catabolism, posttranslational modification, protein turnover, chaperones, replication, recombination and repair, and signal transduction mechanisms increase over those of the model strain *B. subtilis* subsp*. subtilis* str. 168 (Table S1). These categories include a large number of stress response and environmental adaptation proteins, implying that strain DM2 has a strong adaptive capacity to environments.

### 3.4. Whole-Genome Alignments Reveal Heterogeneity within Strains of *B. subtilis*

**Figure 3:**
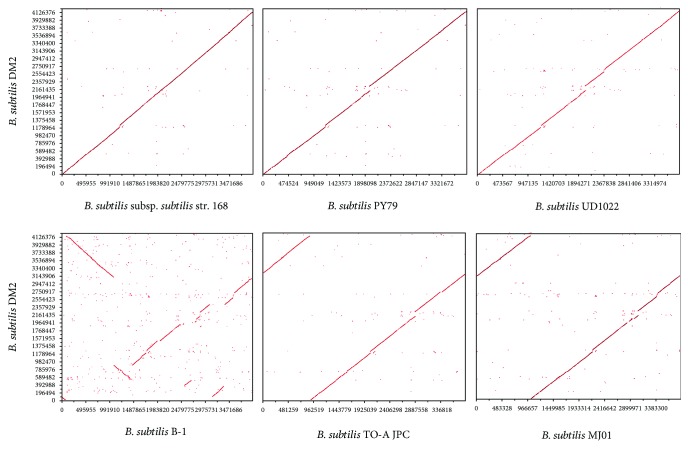
Ortholog dot plot of *B. subtilis* DM2 genome vs. *B. subtilis* PY79, *B. subtilis* subsp*. subtilis* str. 168, *B. subtilis* UD1022, *B. subtilis* B-1, *B. subtilis* TO-A JPC, and *B. subtilis* MJ01 genomes. Each dot represents a reciprocal best hit by BLASTp.

In general, the genetic distance and gene similarity between two organisms can be determined by DNA-DNA hybridization (DDH). Recently, the Genome Blast Distance Phylogeny (GBDP) approach was improved for *in silico* genome-to-genome comparison [27, 28]. The principle of this approach is, firstly, two genomes are aligned using BLAST to generate a set of high-scoring segment pairs, and secondly, a single genome-to-genome distance value is calculated from the total number of identical base pairs by a specific distance formula [28]. The DDH values between the whole genomes of strain DM2 and other *B. subtilis* strains, which have publicly available complete genomes, were calculated using the genome-genome distance calculator (GGDC) server at http://ggdc.dsmz.de [28]. Because the length of high-scoring pairs was used for calculation instead of the genome length, the Formula II values were used as the analysis standards. Strain DM2 was the closest to *B. subtilis* PY79 with 89% DDH value followed by *B. subtilis* NCIB 3610 and *B. subtilis* subsp*. subtilis* str. 168, both with 88.6% DDH value, suggesting that strain DM2 belongs to *B. subtilis* subsp*. subtilis* subspecies. However, DDH values < 70%, which is a threshold for species delimitation in *Archaea* and *Bacteria* [28], for strain DM2 and *B. subtilis* subsp*. stercoris*, *B. subtilis* subsp*. spizizenii*, *B. subtilis* subsp*. inaquosorum*, and *B. subtilis* subsp*. spizizenii* suggest significant genome variations among these *B. subtilis* strains (Table 3). Pairwise genome alignments show that the genomic organization of strain DM2 has high similarity with *B. subtilis* subsp*. subtilis* str. 168, *B. subtilis* PY79, and *B. subtilis* UD1022. No rearrangement is evident, but only a few chromosomal deletions between 1178 and 1375 Kbps are observed in the chromosome. However, chromosomal inversions are observed among strains DM2, *B. subtilis* MJ01, and *B. subtilis* TO-A JPC. Synteny analysis showed various genome rearrangements between strains DM2 and B-1 with numerous genomic insertions, deletions, and inversions (Figure 3). These results indicate that the core genomes of the *Bacillus subtilis* strains are conserved.

### 3.5. Phylogenetic Analysis of *B. subtilis* Strain DM2

**Figure 4:**
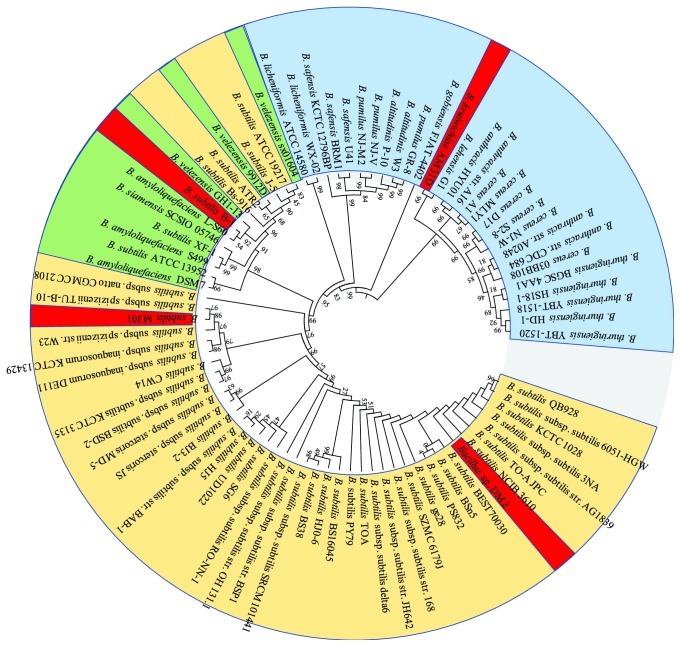
Neighbor-joining phylogenetic tree based on the protein sequences of 24 housekeeping genes of the Bacillaceae family members. The members of *B. subtilis* (orange-colored), outgroups of *B. subtilis* (blue-colored), the members of *B. velezensis* and *B. amyloliquefaciens* (green-colored), and the isolates from the crude-oil environment (red-colored) are included.

To understand the phylogenetic relationship of strain DM2, the protein sequences of 24 housekeeping genes of the members of *B. subtilis* and other *Bacillus* species were aligned using MEGAx. The neighbor-joining phylogenetic tree shows multiple clades (Figure 4). Although the DDH value indicates the closest similarity between the strains DM2 and *B. subtilis* subsp*. subtilis* str. 168, strain DM2 belongs to a separate clade in the phylogenetic tree compared with the other petroleum-degrading strains such as *B. subtilis* MJ01, *B. subtilis* B-1, and *B. krulwichiae*, which are only distantly related. Furthermore, an intermix of strains *B. velezensis* and *B. amyloliquefaciens* within the clades of *B. subtilis* implies that these strains have the closest phylogenetic relationship. In addition to *B. krulwichiae*, *B. pumilus*, and *B. safensis*, which have been reported as isolated from oil-contaminated environments, to date [29–31], most of the oil-degrading *Bacillus* strains belong to *B. subtilis*, suggesting that *B. subtilis* possesses the functional diversity and adaptive capacity to various environments.

### 3.6. Core Proteome Analysis of Strain DM2

**Figure 5:**
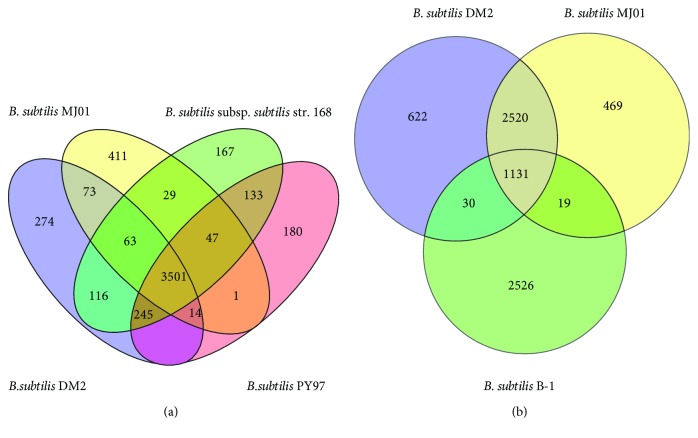
Comparative analysis of protein sets among four *B. subtilis* strains selected. (a) Orthologous set of proteins in *B. subtilis* DM2, *B. subtilis* subsp*. subtilis* str. 168, *B. subtilis* PY79, and *B. subtilis* MJ01. (b) Orthologous set of proteins in *B. subtilis* DM2, *B. subtilis* MJ01, and *B. subtilis* B-1.

The orthologous proteins of four *B. subtilis* strains, which have the closest phylogeny or functional similarity, were aligned using Proteinortho V2.3 Perl script (Figure 5(a)). A total of 3501 proteins formed the core set of proteins of the four strains. Strain DM2 shares 3925, 3777, and 3651 common orthologous proteins with strains *B. subtilis* 168, PY79, and MJ01, respectively. To further unravel the differences in orthologous proteins among the *Bacillus* strains isolated from oil-contaminated environments, the orthologous proteins of strains DM2, MJ01, and B-1 were aligned. The result shows that 1131 orthologous proteins are shared by three strains; 3651 orthologous proteins are shared by strains DM2 and MJ01, but only 1161 orthologous proteins are shared by strains DM2 and B-1 (Figure 5(b)). This result indicates that there are great differences between strains DM2 and B-1, although both are the *B. subtilis* members capable of degrading petroleum. The 1131 orthologous proteins shared with the three strains were further aligned using BLASTp to identify the conserved function genes. The analysis shows that, apart from housekeeping genes, the genes responsible for sporulation/spore germination proteins, chaperones, membrane transport proteins, and transcriptional regulators are the functionally conserved *Bacillus* genes. Moreover, the genes encoding ring-cleaving dioxygenase, fatty acid desaturase, cytochrome P450, oxidoreductase, and FAD-binding oxidoreductase are highly conserved genes in these three petroleum-degrading strains, providing the molecular basis for petroleum biodegradation.

### 3.7. Characterization of Proteins Encoded by Strain DM2 Genome

The above analyses indicate that strain DM2 has the most protein-coding genes among the *B. subtilis* strains compared in this selected panel. Comparison analysis of the identified protein coding regions of strain DM2, model strain 168, and another oil-degrading strain MJ01 shows that strain DM2 shares several common proteins with strain MJ01, including DNA-binding response regulators, EamA family transporters, NAD(P)-dependent oxidoreductases, two-component sensor histidine kinases, two-component system response regulators, and methyl-accepting chemotaxis proteins. Most of these proteins may function in response to stresses and signal transduction. Some of them are also involved in hydrocarbon degradation, such as NAD(P)-dependent alcohol dehydrogenases. Furthermore, some proteins are DM2-specific, such as carboxymuconolactone decarboxylase family, gfo/Idh/MocA family oxidoreductases, GlsB/YeaQ/YmgE family stress response membrane proteins, HlyC/CorC family transporters, LLM class flavin-dependent oxidoreductase, and LPXTG cell wall anchor domain-containing proteins (Table S2). However, most of the abovementioned proteins are absent from strain 168. We conclude that the strain-specific proteins further imply the vigorous adaptability of strain DM2 to the harsh biotope.

### 3.8. Horizontal Gene Transfers in the Genome of Strain DM2

**Figure 6:**
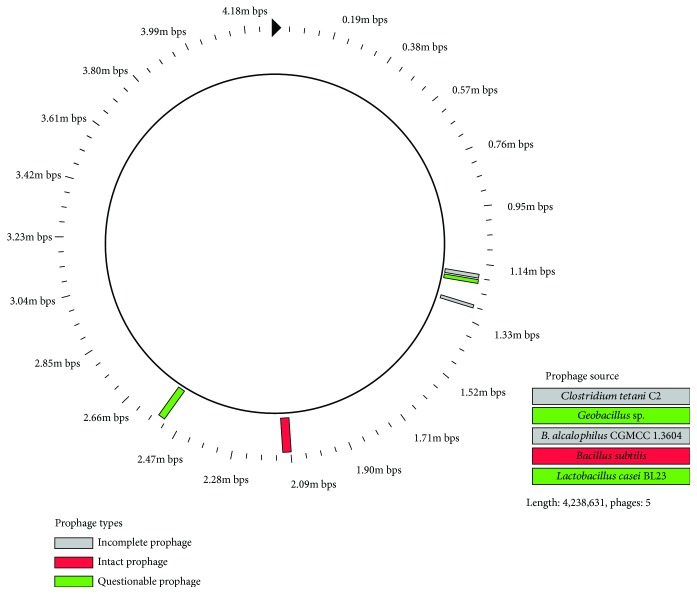
Characteristics and position of the prophage found in the *Bacillus subtilis* DM2 genome. The color in the circles represents the completeness of the prophage, incomplete prophage (gray-colored), intact prophage (red-colored), and potential prophage (green-colored).

The analysis of nonorthologous proteins reveals that the genome contains many horizontal gene transfers. A total of 34 gene islands (GIs) are found in the genome, which consist of 4000 bp-100,000 bp DNA in size (Table 4). Of the total 510 genes, 330 genes are annotated as hypothetical proteins with unknown function, 37 are annotated as recombinase- and phage-related proteins, most of which are the phage-specific site integrases. Most of the genes in GI are associated with metabolism (22), transcriptional regulation (33), signal transduction, and membrane transport (10). Notably, several genes, including glycosyl transferase family A, fatty acid desaturase, short chain dehydrogenase family protein, stress response protein, and cold-shock protein, which are involved in glycosyl transfer, lipid metabolism, and stress response, are found in the GIs. The harboring of these genes in GIs suggests that horizontal gene transfer provides additional clues about metabolic diversity [26] and confers several functional genes to strain DM2 to cope with the harsh environment and to promote petroleum degradation [32]. In addition, a total of 5 prophages are found in the genome of strain DM2, which comprise an intact (123 kb), two incomplete (30 kb), and two questionable (28 kb and 61 kb) prophages (Figure 6). The protein sequences of prophages were further searched in the Nr database using BLASTp, and the result indicates that only an intact prophage protein initiated from *B. subtilis*, whereas the remaining protein sequences evolved from the outgroup prophages of *Bacillus*. The presence of prophages in the genome reflects phage-related genetic modifications and is well-known to regulate bacterial population density. Therefore, the gene transfer, which occurred in strain DM2, plays critical roles in the acquisition of the resistance genes and adaptation to harsh environments [33].

### 3.9. KEGG Pathway Enrichment Reveals the Metabolic Character of Strain DM2

#### 3.9.1. Metabolism

**Figure 7:**
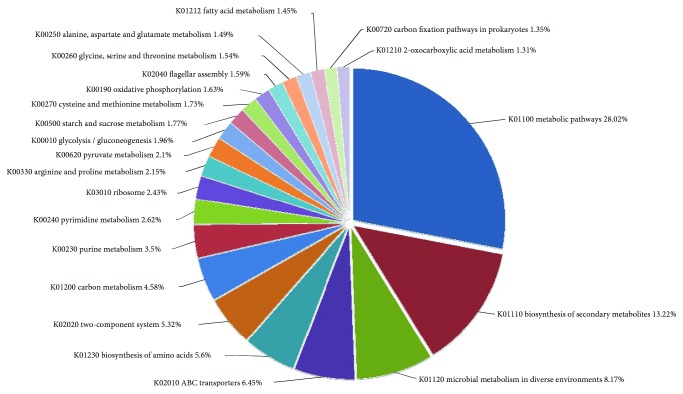
Top KEGG pathways enriched in strain DM2 genome and the percentage of genes under each pathway.

A total of 600 genes are enriched in the metabolic and synthetic pathways, including whole genes associated with glycolysis, TCA cycle, and pentose phosphate pathway, but are lacking the key gene coding for 2-dehydro-3-deoxy-phosphogluconate in Entner-Doudoroff pathway. Strain DM2 can use sucrose, fructose, galactose, rhamnose, mannose, and C_5_-branched dibasic acid as substrates. However, the key genes involved in pathways of fucose, allose, and sorbitol are lacking. The genes involved in polysaccharide metabolism, such as dextranase and amylase genes, are found in strain DM2 genome. All the genes required for the anabolic pathways of amino acids, purines, and pyrimidine synthesis are present in the genome. A total of 18 genes are involved in nitrogen metabolic pathway, of which 7 genes encode nitrite reductase (nirB, nirC, nirD, narG, narH, narI, and narJ) that catalyze nitrate to ammonia. In addition, 2 genes encode nitronate monooxygenases that catalyze nitroalkane to nitrite. The lack of a gene coding for nitrogenase suggests the absence of nitrogen fixation. All key enzymes involved in synthesis of cysteine from sulfate are found in the genome, but the gene coding for sulfate transport system substrate-binding protein (Cysp) is absent. Thus, the presence of genes coding for sulfonate transport protein (ssuA) and alkanesulfonate monooxygenase (ssuD) in the DM2 genome implies that the strain uses alkanesulfonate rather than sulfate as a sulfur supply (Figure 7).

#### 3.9.2. Osmoprotectant Transport Systems

The osmoprotectant transport system (Opu) in the genome of the strain comprises two *opuA* (orf3675 and orf3680), four *opuBD* (orf3672, orf3674, orf3677, and orf3679), and two *opuC* (orf3673 and orf3678) genes. The genes that are involved in the absorption and synthesis of glutamate, which acts as osmoprotectant, include three *gltA* (orf0968, orf2624, and orf3162), a *gltD* (orf2036), a *gltB* (orf2037), a *gltC* (orf2038), and two *gltP/T* (orf0240 and orf1048) genes.

#### 3.9.3. Pathways for Degradation of Petroleum Hydrocarbons and Xenobiotics

Strain DM2 genome harbors a total of 37 genes that may be responsible for hydrocarbon degradation. Of those, 11 genes encode dioxygenases, 13 genes encode monooxygenases, 8 genes encode cytochrome P450 enzymes, and single genes encode fatty acid desaturase, dihydropteridine reductase, and NADH-dependent butanol dehydrogenase. Among them, catechol-2,3-dioxygenase, biphenyl-2,3-dioxygenase, 4-hydroxyphenylacetate 3-monooxygenase, cytochrome P450 CYP102A2_3, and ring-cleaving dioxygenase are the key aromatic degradation enzymes. Some monooxygenases, cytochrome P450 enzymes, NADH-dependent butanol dehydrogenases, fatty acid beta-hydroxylases, and fatty acid desaturases are involved in the degradation pathways of alkanes and alkenes (Table 5). The enzyme fatty acid desaturase is also an important member that plays roles in the adaptability to low temperature [13]. Interestingly, a ubiquitous gene coding for alkane monooxygenase (AlkB) is not found in the genome of strain DM2. Additionally, another gene coding for bacterial luciferase (LadA) involved in the degradation of long-chain hydrocarbon [34], which is harbored in the genomes of strains 168 and MJ01, is not found in DM2 genome.

The xenobiotic biodegradation and metabolism pathways consist of benzoate degradation, aminobenzoate degradation, chloroalkane and chloroalkene degradation, bisphenol degradation, azathioprine and 6-mercaptopurine degradation, fluorouracil degradation, and citalopram degradation (Table 6).

#### 3.9.4. Stress Response and Signaling

The genomic analysis indicates that strain DM2 has developed systems of stress response and signal transduction. Many genes that are related to environmental stress response and signal transduction are found in the genome of strain DM2. A total of 122 genes encode two-component signal transduction proteins, including 19 histidine kinases, 21 response regulators, 11 sporulation and bacteria movement-related genes, and 8 diguanylate cyclases (Table 7). Of those, the histidine kinases act as the stimulus sensor and play a critical role in signal transduction [35]. These histidine kinase genes are adjacent to the genes that code for response regulators in the genome, suggesting that strain DM2 has a highly efficient two-component signaling system and may respond efficiently to the environmental signals [36]. Diguanylate cyclase catalyzes the formation of cyclic diguanylate monophosphate and acts as the ubiquitous secondary messenger involved in various bacterial metabolic and growth processes [37]. The common Sec-dependent secretion system and twin arginine targeting secretion system in the genome are beneficial to the substance exchange across cell membrane and even remold the environment for its growth [38]. In addition, strain DM2 contains 18 chaperone genes, including RNA chaperone, molecular chaperone, nitrate reductase molybdenum cofactor assembly chaperone, copper chaperone, flagellar biosynthesis chaperone, heat shock proteins, and cold shock proteins. Chaperones stabilize the protein conformations and have been shown to contribute to bacterial growth at low temperatures [39]. Strain DM2 possesses two biotin carboxylase genes that have been reported to be expressed at low temperature [40].

#### 3.9.5. ABC Transport Systems

Another prominent feature of the genome of strain DM2 is the powerful membrane transport systems, particularly the ABC transport systems. A total of 310 genes are related to the membrane transport systems. Among them, 138 genes encode the ABC transporters, including ATP-binding protein, substrate-binding proteins, and permeases (Table 8). ABC transporters play important roles in active transmembrane transport, acting as alkanesulfonate transporters, glycine betaine/proline phosphate transporters, amino acid transporters, and osmoprotectants. ABC transporters mediate the transport of glutamine/cystine/D-methionine, maltose/maltodextrin/galactose oligomer, raffinose/stachyose/melibiose, oligopeptide, dipeptide, biotin, and bacitracin, as well as iron complex/iron II, zinc, manganese, and Na^+^ (Table 6). In addition, the DM2 genome contains several phosphotransferase systems (PTS), which is a major carbohydrate active transport system, indicating that these transporters are responsible for the carbohydrate transport into cells.

## 4. Conclusion

The *B. subtilis* strain DM2 isolated from petroleum-contaminated soil on the Tibetan Plateau displays a great capacity to degrade petroleum at a low temperature. The complete genome sequencing and genomic analysis of strain DM2 help us to unravel its biological features that enable it to successfully utilize hydrocarbons as carbon source and potentially withstand other environmental challenges. Strain DM2 is clustered as a separate and a higher evolutionary clade in the phylogenetic tree based on 24 housekeeping protein sequences, implying its unique position with respect to other *B. subtilis* strains. Strain DM2 possesses the largest genome and the most protein-coding genes relative to the other compared *B. subtilis* strains. DDH values show that strain DM2 belongs to *B. subtilis* subsp*. subtilis*, but significant variations in the genome occurred with respect to the other strains or subspecies. Comparative genomic analysis identified the core proteome common to strain DM2, model strain *B. subtilis* subsp*. subtilis* 168, and other *B. subtilis* strains. Strain DM2 possesses almost the same strain-specific proteins as strain MJ01, which is another oil-degrading *B. subtilis* strain, unlike strain *B. subtilis* subsp*. subtilis* 168. Furthermore, many strain DM2-specific proteins were also identified, such as carboxymuconolactone decarboxylase family protein, gfo/Idh/MocA family oxidoreductases, GlsB/YeaQ/YmgE family stress response membrane protein, HlyC/CorC family transporters, LLM class flavin-dependent oxidoreductase, and LPXTG cell wall anchor domain-containing protein. Most of these strain-specific proteins have been shown to be involved in the pathways related to stress response, signaling, and hydrocarbon degradation, suggesting that the main feature of the DM2 genome is the evolutionary occurrence of many genes related to environmental adaptation and carbon utilization. The genomic information provided by the present study might help us to further reveal the genetic and genomic characters of *Bacillus subtilis*, which is a ubiquitous and important bacterial species.

## Figures and Tables

**Table 1:** Genome organization of *B. subtilis* strain DM2.

Genome organization	Chromosome	Plasmid
Genome	1	1
Genome size (bp)	4,238,631	84,240
G+C content (%)	43.52	35.08
Gene number	4458	103
Total gene length (bp)	3,754,593	73,761
Gene average length(bp)	842.21	716.13
Gene density (kb)	1.05	1.22
GC content in gene region (%)	44.18	35.75
Gene/genome (%)	88.58	87.56
Intergenetic region length (bp)	484,038	10,479
GC content in intergenetic region (%)	38.34	30.36
Tandem repeat number	22	-
Tandem repeat length (bp)	6612	-

**Table 2:** Genomic characteristics of several *Bacillus subtilis* strains.

Genome organization	*B. subtilis* strains						
	DM2	PY79	168	UD1022	B-1	MJ01	TO-A JPC
Isolated habitat	Oil field soil	Prototrophic laboratory strain	Hay infusion	Rhizosphere soil	Oil field biofilm	Oil-polluted soil	Probiotic drug
Genome size (Mb)	4.24	4.03	4.22	4.03	3.94	4.11	4.09
GC content (%)	43.5	43.8	43.5	43.9	46.5	43.9	43.8
Total genes	4527	4187	4419	4129	3868	4223	4236
Protein-coding genes	4458	4138	4255	3933	3706	3999	4058
rRNA number	30	30	30	30	19	30	30
tRNA number	88	85	86	86	59	86	86
Other RNA number	5	1	5	1	1	5	5
Pseudogene number	93	2	43	79	83	103	57
NCBI accession	CP030937	CP006881	CP010052	CP011534	CP009684	CP018173	CP011882

**Table 3:** DNA-DNA hybridization values as calculated by GGDC server.

Query genome	Reference genome	Formula I	Formula II	Formula III
*B. subtilis* DM2	*B. subtilis* PY79	92.4	89	94.2
*B. subtilis* DM2	*B. subtilis* subsp*. subtilis* NCIB 3610	95.1	88.6	96.1
*B. subtilis* DM2	*B. subtilis* subsp*. subtilis* str. 168	93.5	88.6	94.9
*B. subtilis* DM2	*B. subtilis* subsp*. subtilis* CU1050	92.9	88.6	94.5
*B. subtilis* DM2	*B. subtilis* subsp*. subtilis* str. AG1839	92.8	88.5	94.4
*B. subtilis* DM2	*B. subtilis* QB928	92.8	88.4	94.4
*B. subtilis* DM2	*B. subtilis* BSn5	92.1	88.4	93.9
*B. subtilis* DM2	*B. subtilis* subsp*. subtilis* str. OH 131.1	92.2	87.9	93.9
*B. subtilis* DM2	*B. subtilis* UD1022	91.4	85.3	92.9
*B. subtilis* DM2	*B. subtilis* subsp*. subtilis* RO-NN-1	89.5	83.3	91.2
*B. subtilis* DM2	*B. subtilis* subsp*. stercoris* JS	88.4	62.9	86.4
*B. subtilis* DM2	*B. subtilis* subsp*. stercoris* MD-5	87.9	62.7	85.9
*B. subtilis* DM2	*B. subtilis* MJ01	84.5	51.2	79.7
*B. subtilis* DM2	*B. subtilis* subsp*. spizizenii* TU-B-10	83.9	50.5	79
*B. subtilis* DM2	*B. subtilis* subsp*. inaquosorum* KCTC 13429	81.1	50.5	76.6
*B. subtilis* DM2	*B. subtilis* subsp*. inaquosorum* DE111	80	50.3	75.7
*B. subtilis* DM2	*B. subtilis* subsp*. spizizenii* W23	83	49.5	77.9
*B. subtilis* DM2	*B. vallismortis* NBIF-001	33.1	21	28.9
*B. subtilis* DM2	*B. velezensis* FZB42	33	20.9	28.7
*B. subtilis* DM2	*B. subtilis* J-5	32.4	20.9	28.4
*B. subtilis* DM2	*B. subtilis* B-1	32	20	28

**Table 4:** Features of the gene islands found in the genome of *B. subtilis* DM2.

GI	Size (bp)	Genes	Hypothetical proteins	Integrase/phage portal protein
1	10,848	9	3	1
2	4247	3	1	0
3	10,269	4	3	0
4	11,092	4	4	0
5	7217	7	4	0
6	8419	8	5	1
7	26,611	26	13	1
8	4522	5	4	0
9	5365	5	2	0
10	4179	7	2	2
11	4778	11	5	1
12	5696	7	4	0
13	18,793	27	18	2
14	6295	3	2	0
15	7427	11	7	0
16	122,642	164	122	5
17	4157	7	5	0
18	6329	14	10	0
19	24,186	41	32	2
20	4642	9	8	0
21	8129	7	7	0
22	5209	9	8	1
23	14,435	7	6	0
24	6531	9	4	1
25	40,710	53	23	19
26	4006	8	6	1
27	6429	8	2	0
28	4134	2	2	0
29	5506	7	1	0
30	4168	2	0	0
31	4087	6	5	0
32	7752	7	4	0
33	6928	7	4	0
34	4389	6	4	0
Total	420,127	510	330	37

**Table 5:** List of proteins involve in degradation of petroleum compounds.

Protein ID	Swissprot references	Swissprot closest homolog	Swissprot similarity %	KO	Gene name/enzyme
AXF32711	spo31669.1	Acireductone dioxygenase	100	K08967	mtnD, mtnZ, ADI1
AXF32173	spp40402.2	Alkanesulfonate monooxygenase	100	K04091	ssuD
AXF32337	spo07561.1	Aromatic compound monooxygenase	99	None	_
AXF34322	spo34627.1	Blue-light photoreceptor	99	None	_
AXF32118	spP54720.1	Catechol-2,3-dioxygenase	99	None	_
AXF32119	spp54721.2	Catechol-2,3-dioxygenase	99	K07104	_
AXF34401	spo32085.1	Cysteine dioxygenase	97	None	_
AXF35528	spp53554.1	Cytochrome P450	98	K16593	bioI
AXF34015	spo08336.1	Cytochrome P450 CYP102A3	99	K14338	cypD_E, CYP102A2_3
AXF31825	spo34653.1	Fatty acid desaturase	82	K10255	FAD6, desA
AXF33257	spo34653.1	Fatty acid desaturase	100	K10255	FAD6, desA
AXF31559	spo31440.1	Fatty acid peroxygenase	100	K15629	CYP152A1
AXF32654	spp49852.1	Flavohemoprotein	100	K05916	hmp, YHB1
AXF32298	spp38049.1	Heme-degrading monooxygenase	99	None	_
AXF35412	spo31534.2	Heme-degrading monooxygenase	100	K07145	isdG, isdI
AXF33211	spc0SPC0.1	Probable 4-hydroxyphenylacetate 3-monooxygenase	100	K00483	hpaB
AXF34426	spo05239.1	Probable NADH-dependent butanol dehydrogenase 1	100	K00100	E1.1.1.-
AXF34427	spo05240.1	Probable NADH-dependent butanol dehydrogenase 2	100	None	_
AXF33973	spo05413.1	Probable nitronate monooxygenase	99	K00459	ncd2, npd
AXF32025	spo08394.1	Putative cytochrome P450 CYP102A2	99	K14338	cypD_E, CYP102A2_3
AXF32565	spo34374.1	Putative cytochrome P450 YjiB	99	K00517	E1.14.-.-
AXF32015	spo31535.1	Putative lyase/dioxygenase	100	None	_
AXF31633	spo34504.2	Putative monooxygenase	99	None	_
AXF31730	spp94425.1	Putative monooxygenase	100	None	_
AXF34230	spo34846.1	Putative monooxygenase	99	None	_
AXF35096	spp39606.1	Putative monooxygenase	99	None	_
AXF35236	spp54950.1	Putative monooxygenase	100	None	_
AXF34689	spo32254.1	Putative monooxygenase	100	None	_
AXF34228	spo34974.1	Putative monooxygenase MoxC	99	None	_
AXF31862	spp96693.1	Putative ring-cleaving dioxygenase	100	K15975	_
AXF32639	spo34689.1	Putative ring-cleaving dioxygenase MhqA	100	K15975	_
AXF33297	spo34543.1	Putative ring-cleaving dioxygenase MhqE	99	K15975	_
AXF35280	spp42106.2	Quercetin 2,3-dioxygenase	99	K07155	E1.13.11.24

**Table 6:** List of KO pathways for hydrocarbon and aromatic compound degradation.

KO	Genes	Enzymes	Protein ID
K00121	*frmA*, *ADH*, *adhC*	Alcohol dehydrogenase (EC: 1.1.1.1)	AXF33996
K00128	*ALDH*	Aldehyde dehydrogenase family protein (EC: 1.2.1.3)	AXF35274, AXF35172
			AXF35083, AXF33272
			AXF32034, AXF31595
K00493	*CYP*	Cytochrome P450 (EC: 1.14.14.1)	AXF32025
K00626	*atoB*	Acetyl-CoA C-acetyltransferase (EC: 2.3.1.9)	AXF32322, AXF33714
K00074	*paaH*	3-Hydroxybutyryl-CoA dehydrogenase (EC: 1.1.1.157)	AXF33713
K01670	*nmsA*	Naphthyl-2-methylsuccinate synthase alpha subunit (EC: 4.1.99.-)	AXF34000
K00632	*fadA/I*	Acetyl-CoA acyltransferase (EC: 2.3.1.16)	AXF34569
K01821	*praC*	4-Oxalocrotonate tautomerase (EC: 5.3.2.6)	AXF35041
K01607	*pcaC*	4-Carboxymuconolactone decarboxylase (EC: 4.1.1.44)	AXF35133
K01113	*phoD*	Alkaline phosphatase D (EC: 3.1.3.1)	AXF31608
K01077	*phoA*	Alkaline phosphatase (EC: 3.1.3.1)	AXF31889
			AXF32230
K01512	*acyP*	Acylphosphatase (EC: 3.6.1.7)	AXF32061
K00517	*CYP81F*	Cytochrome P450 (EC: 1.14.14.-)	AXF32565
K01034	*atoD*	Acetate CoA/acetoacetate CoA-transferase alpha subunit (EC: 2.8.3.8)	AXF33316
K01101	*-*	4-Nitrophenyl phosphatase (EC: 3.1.3.41)	AXF34513
K00100	*bdhAB*	Butanol dehydrogenase (EC: 1.1.1.-)	AXF34427
K00148	*fdhA*	Glutathione-independent formaldehyde dehydrogenase (EC: 1.2.1.46)	AXF35308
K01428	*ureC*	Urease subunit alpha (EC: 3.5.1.5)	AXF34944
K01429	*ureB*	Urease subunit beta (EC: 3.5.1.5)	AXF34945
K01430	*ureA*	Urease subunit gamma (EC: 3.5.1.5)	AXF34946

**Table 7:** The two-component signaling systems of *B. subtilis* DM2.

Gene families	Genes	Description	KOs	Protein ID
LytTR	*natK*	Sensor histidine kinase	K11640	AXF31619
	*natR*	Response regulator	K11641	AXF31620
	*lytT/lytR*	Response regulator	K07705	AXF34190
	*lytS*	Sensor histidine kinase	K07704	AXF34191

CitB	*dctS*	Sensor histidine kinase	K11691	AXF31786
	*dctR*	Response regulator	K11692	AXF31787
	*citS*	Sensor histidine kinase	K11637	AXF32055
	*citT*	Response regulator	K11638	AXF32056
	*yufL/malK*	Sensor histidine kinase	K11614	AXF34443
	*malR*	Response regulator	K11615	AXF34444

NarL	*ydfH*	Sensor histidine kinase	K11623	AXF31854
	*ydfI*	Response regulator	K11624	AXF31855
	*desK*	Sensor histidine kinase	K07778	AXF33258
	*desR*	Response regulator	K07693	AXF33259
	*comA*	Two-component response regulator	K07691	AXF34459
	*comP*	Sensor histidine kinase	K07680	AXF34460
	*liaR*	Response regulator	K11618	AXF34595
	*liaS*	Sensor histidine kinase	K11617	AXF34596
	*desR*	Response regulator	K07693	AXF34697
	*desK*	Sensor histidine kinase	K07778	AXF35550
	*degU*	Response regulation	K07692	AXF34832
	*degS*	Sensor histidine kinase	K07777	AXF34833

OmpR	*resE*	Sensor histidine kinase	K07651	AXF33616
	*resD*	Response regulator	K07775	AXF33617
	*phoR*	Phosphate regulon histidine kinase	K07636	AXF34207
	*phoB1/phoP*	Alkaline phosphatase synthesis response regulator	K07658	AXF34208
	*bceS*	Bacitracin resistance histidine kinase	K11629	AXF34327
	*bceR*	Bacitracin resistance response regulator	K11630	AXF34328
	*cssR*	Response regulator	K07770	AXF34586
	*cssS*	Sensor histidine kinase	K07650	AXF34587
	*yxdK*	Sensor histidine kinase	K11633	AXF35248
	*yxdJ*	Response regulator	K11634	AXF35249
	*vicK*	Sensor histidine kinase	K07652	AXF35323
	*vicR*	Response regulator	K07668	AXF35575

Unknown	*ycbA/glnK*	Sensor histidine kinase	K07717	AXF35385
	*ycbB/glnL*	Response regulator	K07719	AXF31592
	*yesM*	Sensor histidine kinase	K07718	AXF31995
	*yesN*	Response regulator	K07720	AXF31996

**Table 8:** The ABC transport systems in the genome of *B. subtilis* DM2.

Genes	Description	KOs	Protein ID
*ssuA*	Sulfonate transport system substrate-binding protein	K15553	AXF32171
*ssuC*	Sulfonate transport system permease protein	K15554	AXF32172
*ssuB*	Sulfonate transport system ATP-binding protein	K15555	AXF32170
*proX*	Glycine betaine/proline transport substrate-binding protein	K02002	AXF31645
*proW*	Glycine betaine/proline transport permease protein	K02001	AXF31644
*proV*	Glycine betaine/proline transport ATP-binding protein	K02000	AXF31643
*msmE*	Raffinose/stachyose/melibiose substrate-binding protein	K10117	AXF34316/AXF34543
*msmF*	Raffinose/stachyose/melibiose transport permease protein	K10118	AXF34317/AXF34542
*msmG*	Raffinose/stachyose/melibiose transport permease protein	K10119	AXF34318/AXF34541
*msmK*	Multiple sugar transport system ATP-binding protein	K10112	AXF34539/AXF35170
*rbsB*	Ribose transport system substrate-binding protein	K10439	AXF34880
*rbsC*	Ribose transport system permease protein	K10440	AXF34879
*rbsD*	D-Ribose pyranase	K06726	AXF34877
*rbsA*	Ribose transport system ATP-binding protein	K10441	AXF34878
*pstS*	Phosphate transport system substrate-binding protein	K02040	AXF33794
*pstC*	Phosphate transport system permease protein	K02037	AXF33793
*pstA*	Phosphate transport system permease protein	K02038	AXF33792
*pstB*	Phosphate transport system ATP-binding protein	K02036	AXF33791
*yxeM*	Amino acid transport system substrate-binding protein	K16961	AXF35234
*yxeN*	Amino acid transport system permease protein	K16962	AXF35233
*yxeO*	Amino acid transport system ATP-binding protein	K16963	AXF35232
*fhuD*	Iron complex transport system substrate-binding protein	K02016	AXF31727/AXF32049/AXF34604/AXF34618
*fhuB*	Iron complex transport system permease protein	K02015	AXF31724/AXF31725/AXF32047/AXF32048/AXF34603/AXF34617
*fhuC*	Iron complex transport system ATP-binding protein	K02013	AXF31726/AXF32046/AXF35542/AXF34616
*znuA*	Zinc transport system substrate-binding protein	K09815	AXF31630/AXF33983
*znuB*	Zinc transport system permease protein	K09816	AXF31632
*znuC*	Zinc transport system ATP-binding protein	K09817	AXF31631
*troA*	Manganese/zinc/iron transport substrate-binding protein	K11707	AXF34365
*troC*	Manganese/zinc/iron transport system permease protein	K11708	AXF34363
*troD*	Manganese/zinc/iron transport system permease protein	K11709	AXF34362
*troB*	Manganese/zinc/iron transport system ATP-binding protein	K11710	AXF34364
*bioY*	Biotin transport system substrate-specific component	K03523	AXF32324/AXF34491
*ecfT*	Energy-coupling factor transport system permease protein	K16785	AXF31508/AXF32671
*ecfA1*	Energy-coupling factor transport ATP-binding protein	K16786	AXF31506
*ecfA2*	Energy-coupling factor transport ATP-binding protein	K16787	AXF31507/AXF32672
*natB*	Sodium transport system permease protein	K09696	AXF31622
*natA*	Sodium transport system ATP-binding protein	K09697	AXF31621
*bceB*	Bacitracin transport system permease protein	K11632	AXF34325
*bceA*	Bacitracin transport system ATP-binding protein	K11631	AXF34326

## Data Availability

The data used to support the findings of this study are included within the article.

## Supplementary Materials

Supplementary Materials Supplementary Table S1: comparative analysis of COG categories between strain DM2 and strain 168. Supplementary Table S2: list of the strain DM2-specific proteins versus strains PY97, 168, and MJ01.
