# Supplementary material for: Comparative Genome Characterization of a Petroleum-Degrading Bacillus subtilis Strain DM2
Source: Int J Genomics. 2019 May 8;2019:7410823. doi: 10.1155/2019/7410823 (PMC6530121; doi:10.1155/2019/7410823)
Supplement: Supplementary Materials — Supplementary Table S1: comparative analysis of COG categories between strain DM2 and strain 168. Supplementary Table S2: list of the strain DM2-specific proteins versus strains PY97, 168, and MJ01. [file 7410823.f1.pdf]

Supplementary materials

Table S1 Comparative analysis of COG categories between strain DM2 and strain 168

COG categories		Percentage of proteins %		
		Strain DM2	Strain 168	Strain DM2/strain 168
B	Chromatin structure and dynamics	0.03	0.03	0.00
C	Energy production and conversion	4.89	5.08	-3.74
D	Cell cycle control, cell division, chromosome partitioning	1.61	1.64	-1.83
E	Amino acid transport and metabolism	8.94	9.04	-1.11
F	Nucleotide transport and metabolism	2.98	2.95	1.02
G	Carbohydrate transport and metabolism	8.76	8.77	-0.11
H	Coenzyme transport and metabolism	5.48	5.69	-3.69
I	Lipid transport and metabolism	4.02	3.87	3.88
J	Translation, ribosomal structure and biogenesis	6.29	6.39	-1.56
K	Transcription	8.4	8.4	0.00
L	Replication, recombination and repair	3.81	3.71	2.70
M	Cell wall/membrane/envelope biogenesis	5.72	5.69	0.53
N	Cell motility	1.82	1.83	-0.55
O	Posttranslational modification, protein turnover, chaperones	3.72	3.62	2.76
P	Inorganic ion transport and metabolism	5.33	5.36	-0.56
Q	Secondary metabolites biosynthesis, transport and catabolism	2.95	2.86	3.15
R	General function prediction only	8.67	8.86	-2.14
S	Function unknown	6.73	6.76	-0.44
T	Signal transduction mechanisms	5.07	4.96	2.22
U	Intracellular trafficking, secretion, and vesicular transport	0.89	0.91	-2.20
V	Defense mechanisms	2.74	2.71	1.11
W	Extracellular structures	0.12	0.09	33.33

Table S2 List of strain DM2-specific proteins versus strains PY97, 168, and MJ01

Locus	Proteins	PY97	168	MJ01
AXF31570	ADP-ribosylglycohydrolase family protein	-	-	-
AXF31571	TopI - like protein YobL	-	-	-
AXF31819	DUF4365 domain-containing protein	-	-	-
AXF31820	SMI1/KNR4 family protein	-	-	-
AXF31827	DMT family transporter	-	-	-
AXF31940	RNA polymerase	-	-	-
AXF31941	DUF4179 domain-containing protein	-	-	-
AXF32040	peptidase S8	-	+	+
AXF32115	peptidase G2	-	+	-

AXF32201	DUF2812 domain-containing protein	-	-	-
AXF32202	PadR family transcriptional regulator	-	+	+
AXF32207	LPXTG cell wall anchor domain-containing protein	-	-	-
AXF32342	gfo/Idh/MocA family oxidoreductase	-	-	-
AXF32475	site-specific integrase	-	-	+
AXF32479	XRE family transcriptional regulator	-	+	+
AXF32480	XRE family transcriptional regulator	-	+	+
AXF32481	DNA-binding protein	-	+	+
AXF32482	phage regulatory protein	-	-	-
AXF32483	XRE family transcriptional regulator	-	+	+
AXF32501	Fis family transcriptional regulator	-	-	+
AXF32508	PBSX family phage terminase large subunit	-	-	-
AXF32509	phage portal protein	-	+	+
AXF32510	DUF4355 domain-containing protein	-	-	-
AXF32511	P22 coat protein - protein 5 domain protein	-	-	-
AXF32517	DUF3383 family protein	-	-	-
AXF32518	DUF3277 domain-containing protein	-	-	-
AXF32520	LysM domain-containing protein	-	-	-
AXF32524	DUF2634 domain-containing protein	-	-	-
AXF32525	baseplate J/gp47 family protein	-	-	-
AXF32528	XkdX family protein	-	-	-
AXF32530	phage holin	-	-	+
AXF32531	N-acetylmuramoyl-L-alanine amidase	-	+	+
AXF32536	DUF4065 domain-containing protein	-	-	-
AXF32537	YolD-like family protein	-	-	-
AXF32567	DNA adenine methylase	-	-	-
AXF32619	lytic transglycosylase	-	+	+
AXF32749	dipeptide epimerase	-	-	+
AXF33005	ABC transporter ATP-binding protein	-	+	+
AXF33204	sugar porter family MFS transporter	-	-	-
AXF33205	gfo/Idh/MocA family oxidoreductase	-	-	-
AXF33206	AP endonuclease	-	-	-
AXF33207	LacI family transcriptional regulator	-	+	+
AXF33239	tetratricopeptide repeat protein	-	-	-
AXF33328	DUF559 domain-containing protein	-	-	-
AXF33330	small acid-soluble spore protein	-	-	-
AXF33336	DUF1643 domain-containing protein	-	-	-
AXF33338	VanZ family protein	-	-	-
AXF33341	class 1b ribonucleoside-diphosphate reductase	-	-	+
AXF33348	DUF1653 domain-containing protein	-	-	-
AXF33357	DNA cytosine methyltransferase	-	-	-
AXF33358	HNH endonuclease	-	+	-
AXF33457	phage tail protein	-	-	+
AXF33463	phage holin	-	-	+

AXF33464	tetratricopeptide repeat protein	-	-	-
AXF33465	aspartate phosphatase	-	+	+
AXF33470	SMI1/KNR4 family protein	-	-	-
AXF33472	SMI1/KNR4 family protein	-	-	-
AXF33770	HlyC/CorC family transporter	-	-	-
AXF33897	putative holin-like toxin	-	-	-
AXF33932	recombinase	-	+	-
AXF33947	sulfurtransferase	-	+	-
AXF33976	GapA-binding peptide SR1P	-	-	-
AXF33977	MFS transporter	-	+	+
AXF33978	3-oxoacyl-ACP reductase FabG	-	-	-
AXF33979	transketolase family protein	-	-	-
AXF33981	MFS transporter	-	+	+
AXF33982	MarR family transcriptional regulator	-	+	+
AXF34228	LLM class flavin-dependent oxidoreductase	-	-	-
AXF34461	competence pheromone ComX	-	-	-
AXF34462	isoprenyl transferase	-	-	+
AXF34664	gallidermin/nisin family lantibiotic	-	-	-
AXF34665	Subtilin biosynthesis protein SpaC	-	-	-
AXF34666	ABC transporter ATP-binding protein	-	+	+
AXF34667	Subtilin biosynthesis protein SpaB	-	-	-
AXF34680	zinc-ribbon domain-containing protein	-	-	-
AXF35017	bacteriocin-like protein SboX	-	-	-
AXF35033	ABC transporter ATP-binding protein	-	+	+
AXF35132	XRE family transcriptional regulator	-	+	+
AXF35133	carboxymuconolactone decarboxylase family	-	-	-
AXF35134	MFS transporter	-	+	+
AXF35214	DUF4176 domain-containing protein	-	-	-
AXF35268	bacteriocin, lactococcin A1 family protein	-	-	-
AXF35269	bacteriocin, lactobin A family protein	-	-	-
AXF35270	peptide cleavage/export ABC transporter	-	-	-
AXF35271	HlyD family efflux transporter periplasmic adaptor subunit	-	-	-
AXF35342	MerR family transcriptional regulator	-	+	+
AXF35401	DUF3427 domain-containing protein	-	-	-
AXF35407	abhydrolase domain-containing 18	-	-	-
AXF35466	XRE family transcriptional regulator	-	+	+
AXF35483	site-specific integrase	-	-	+
AXF35511	FadR family transcriptional regulator	-	-	-
AXF35570	GlsB/YeaQ/YmgE family stress response membrane protein	-	-	-
DS740_01150	pseudo	-	-	-

Note: + indicates the existence of this gene in the strain; - indicates the lack of this gene in the strain.
